# Factors affecting infection of corals and larval oysters by *Vibrio coralliilyticus*

**DOI:** 10.1371/journal.pone.0199475

**Published:** 2018-06-19

**Authors:** Blake Ushijima, Gary P. Richards, Michael A. Watson, Carla B. Schubiger, Claudia C. Häse

**Affiliations:** 1 Oregon State University, Carlson College of Veterinary Medicine, Corvallis, Oregon, United States of America; 2 United States Department of Agriculture, Agricultural Research Service, Dover, Delaware, United States of America; Bigelow Laboratory for Ocean Sciences, UNITED STATES

## Abstract

The bacterium *Vibrio coralliilyticus* can threaten vital reef ecosystems by causing disease in a variety of coral genera, and, for some strains, increases in virulence at elevated water temperatures. In addition, strains of *V*. *coralliilyticus* (formally identified as *V*. *tubiashii*) have been implicated in mass mortalities of shellfish larvae causing significant economic losses to the shellfish industry. Recently, strain BAA-450, a coral pathogen, was demonstrated to be virulent towards larval Pacific oysters (*Crassostrea gigas*). However, it is unclear whether other coral-associated *V*. *coralliilyticus* strains can cause shellfish mortalities and if infections are influenced by temperature. This study compared dose dependence, temperature impact, and gross pathology of four *V*. *coralliilyticus* strains (BAA-450, OCN008, OCN014 and RE98) on larval *C*. *gigas* raised at 23°C and 27°C, and evaluated whether select virulence factors are required for shellfish infections as they are for corals. All strains were infectious to larval oysters in a dose-dependent manner with OCN014 being the most pathogenic and BAA-450 being the least. At 27°C, higher larval mortalities (*p* < 0.05) were observed for all *V*. *coralliilyticus* strains, ranging from 38.8−93.7%. Gross pathological changes to the velum and cilia occurred in diseased larvae, but there were no distinguishable differences between oysters exposed to different *V*. *coralliilyticus* strains or temperatures. Additionally, in OCN008, the predicted transcriptional regulator ToxR and the outer membrane protein OmpU were important for coral and oyster disease, while mannose sensitive hemagglutinin type IV pili were required only for coral infection. This study demonstrated that multiple coral pathogens can infect oyster larvae in a temperature-dependent manner and identified virulence factors required for infection of both hosts.

## Introduction

Elevated sea surface temperatures caused by global climate change have and will continue to negatively impact the environment. One consequence is the increased frequency of infections caused by bacteria belonging to the genus *Vibrio*, which are responsible for diseases affecting marine organisms and humans [1–9]. One bacterial pathogen, *Vibrio coralliilyticus*, has been associated with disease of the tropical corals *Pocillopora damicornis*, *Acropora cytherea*, *Montipora capitata*, *Montipora aequituberculata*, and *Pachyseris speciosa* [10–12]. In fact, *V*. *coralliilyticus* has been implicated in coral diseases across numerous reefs ranging from the Indo-Pacific, the Great Barrier Reef, Micronesia, Polynesia, the Caribbean, and the Mediterranean [11–15]. Multiple genera of coral are susceptible to infections by *V*. *coralliilyticus*, which includes reef-building species that make up substantial proportions of their native reefs [11,12,15]. Mass coral mortalities can have extensive negative impacts on marine biodiversity [16,17], while the industries they support are worth billions of US dollars [18–20].

Some strains of *V*. *coralliilyticus* disrupt the symbiotic relationship between the coral animal (polyp) and their symbiotic dinoflagellates (*Symbiodinium* spp.), which reside primarily within the gastrodermal cells of the polyp [13,21,22]. The loss of these dinoflagellates, referred to as bleaching, can lead to the death of the coral animal if the algae are not reestablished because they provide the majority of the energy for many coral species through photosynthesis [23,24]. Additionally, increasing sea surface temperatures (≥27°C) appear to enhance the virulence of some *V*. *coralliilyticus* strains causing coral bleaching and death. Ben-Haim et al. (2003) demonstrated that *V*. *coralliilyticus* BAA-450 (also referred to as YB1 in the literature) caused coral bleaching in *Pocillopora damicornis* at 24.5−29.0°C, but not at 20.0−22°C; while tissue lysis occurred at temperatures ≥ 27.0°C [21]. Vidal-Dupiol et al. (2011) also showed the ability of BAA-450 to induce coral bleaching and tissue lysis in *P*. *damicornis* when water temperatures were increased from 25°C to 32.5°C, but not when the temperature remained at 25°C [22].

Another strain of *V*. *coralliilyticus* known as OCN014 was obtained from diseased *Acropora cytherea* from Palmyra Atoll in the Northern Line Islands located in the Central Pacific Ocean [15]. Like BAA-450, OCN014 was virulent at elevated temperatures causing acute tissue loss, but became avirulent at 23°C. Interestingly, OCN014 was not observed to cause bleaching like BAA-450. In contrast to these temperature-dependent strains, mortalities caused by strain OCN008, a cause of acute tissue loss lesions in the Hawaiian coral *Montipora capitata*, did not significantly change over the temperature range of 23 to 27°C [12]. This discrepancy in response to temperature between different strains of *V*. *coralliilyticus* has been attributed to temperature-induced differential expression of virulence factors [15,25,26].

The virulence of *V*. *coralliilyticus* may be regulated by a host of genes including those that influence chemotaxis/motility, host degradation (like proteases, hemolysins/cytolysins, and toxins), resistance factors, secretion systems, and regulatory processes, including quorum sensing, which can be influenced by water temperature [21,25–27]. Relatively few virulence factors have been identified in *V*. *coralliilyticus* and have been linked to diseases of coral [15,21,27]. In one of those studies investigating OCN014 and OCN008, genes encoding the membrane-bound protein, ToxR, and the mannose-sensitive hemagglutinin (MSHA) type IV pili were essential for wild-type levels of coral infection for both strains [15]. These two virulence factors were identified using a genetic screen to discover genes upregulated at 29°C versus 23°C in OCN014. Interestingly, expression of *toxR* and the MSHA-encoding gene cluster did not respond to temperature in OCN008 [15]. The MSHA pili are likely adhesins which are important for *V*. *coralliilyticus* virulence in corals, while ToxR is a predicted regulatory protein that is essential for virulence in a range of different pathogenic vibrios [28–32]. In the human pathogen *Vibrio cholera*, ToxR regulates the expression of the main virulence factors cholerae toxin and the toxin-coregulated pili [28,29]. Additionally, in several pathogenic vibrios, ToxR positively regulates the expression of the outer membrane protein OmpU, which is a predicted adherence factor for *V*. *cholerae* and *V*. *vulnificus* in humans and for *V*. *tasmaniensis* to oyster hemocytes [33–36]. It is not known if these coral-associated virulence factors are also required for infection of the other invertebrate hosts susceptible to *V*. *coralliilyticus* infections, mainly shellfish larvae.

Recently, it was demonstrated that the *V*. *coralliilyticus* type strain BAA-450, an identified coral pathogen, also caused mortalities in larval oysters [37]. Intermittent outbreaks of larval shellfish mortalities have plagued shellfish hatcheries, particularly along the U.S. West Coast, and have been attributed to *V*. *coralliilyticus* (previously misidentified as *Vibrio tubiashii* in the early literature) [37–39]. In 2007, a severe mortality event along the U.S. Pacific Coast resulted in a reported 59% reduction in production at a major hatchery [39]. Such episodes can be common in many hatcheries with some reporting larval mortalities as high as 80%. These extensive mortalities have subsequently led to shortages of seed oysters needed for commercial shellfish operations [37,39]. Although Pacific oysters are susceptible to *V*. *coralliilyticus* infections, a range of other shellfish species are also apparently vulnerable, including the Eastern oyster (*Crassostrea virginica*), Kumamoto oyster (*Crassostrea sikamea*), geoduck clam (*Panopea generosa*), and the Greenshell mussel (*Perna canaliculus*) [37–40]. This relatively broad host-range demonstrates the destructive potential of this pathogen. Though, it is unclear whether *V*. *coralliilyticus* strains possess a single repertoire of virulence factors capable of causing disease in multiple hosts, if there are different sets of virulence factors for different hosts, or if each strain contains different virulence factors which are specific to certain hosts. Infection of shellfish larvae by coral-associated strains and a similar response to temperature would suggest an overlap of virulence factors.

In the present study, the virulence of multiple *V*. *coralliilyticus* strains was tested in a Pacific oyster larvae infection model, which included the coral pathogens OCN008, OCN014, and BAA-450. Additionally, the oyster pathogen *V*. *coralliilyticus* strain RE98 (hereafter RE98) [38] was included in this study to compare this strain, isolated from diseased *C*. *gigas* larvae, to the coral-associated strains. Multiple *V*. *coralliilyticus* strains were investigated to determine if the coral-associated strains are pathogenic to *C*. *gigas*, as well as to assess if the regulation of virulence is conserved between these strains. Specifically, we: (i) compared different doses of OCN014, OCN008, BAA-450, and RE98 in a *C*. *gigas* larvae infection model, (ii) determined if elevated seawater temperatures caused increased mortalities in larval oysters, (iii) determined whether a deletion mutant deficient in MSHA is required for infection of larval oysters like it is in corals, and (iv) evaluated whether genes encoding ToxR or the predicted porin protein OmpU are involved with pathogenicity in larval oysters as they are in corals.

## Materials and methods

### Bacterial strains and growth conditions

All bacterial strains used in this study are listed in Table 1. Marine bacteria were grown in a modified glycerol artificial seawater (GASW) medium [41] and incubated at 23 or 27°C corresponding to the temperature of the subsequent infection experiment. For plates or overnight growth, the GASW medium was supplemented with 50 mM Tris-Base (GASW-Tris) and the pH adjusted to 8.3 using HCl. Thiosulfate citrate bile salts sucrose (TCBS) agar (Sigma-Aldrich, St. Louis, MO) was prepared according to the manufacturer’s instructions but was supplemented with NaCl for a final concentration of 2.0% (w/v). Unless otherwise stated, all media were incubated at 27°C for the growth of *Vibrio* strains, all *E*. *coli* strains were grown in LB-Miller (Sigma-Aldrich) at 37°C and antibiotics for selection with *E*. *coli* were used at the following concentrations: kanamycin, 50 μg/ml; streptomycin, 25 μg/ml; spectinomycin, 50 μg/ml; and chloramphenicol, 15 μg/ml. Antibiotics for selection with *V*. *coralliilyticus* were used at the following concentrations, unless otherwise stated: ampicillin, 200 μg/ml; streptomycin, 60 μg/ml; spectinomycin, 100 μg/ml; and chloramphenicol, 10 μg/ml. Growth media for *E*. *coli* auxotrophic strains were supplemented with deoxythymidine (DT) or diaminopimelate (DAP) at a final concentration of 0.3 mM as required. Arabinose-inducible expression of the *ccdB* gene was achieved by the addition of 0.3% L-arabinose to GASW-Tris (GASW-ARA) and expression was repressed by the addition of 1% D-glucose to LB-Miller (LB-DEX) or GASW-Tris (GASW-DEX) broth [42]. *Vibrio* cultures were washed with artificial seawater (ASW), which is GASW lacking glycerol, tryptone, and yeast extract, while phosphate buffered saline (PBS) was used for *E*. *coli* strains. *Vibrio* sp. HMSC5 (hereafter HMSC5) was provided by Dr. Christopher Langdon, Oregon State University (OSU), Hatfield Marine Science Center, Newport, OR. It was isolated from tank water containing *C*. *gigas* spat that had been plated onto TCBS agar. HMSC5 was previously determined to be non-pathogenic to *C*. *gigas* larvae (Langdon, pers. comm.). *Vibrio tasmaniensis* strain LGP32 was kindly provided by Dr. Frédérique Le Roux, Institut Français de Recherche pour l'Exploitation de la Mer (IFREMER), La Tremblade, France.

**Table 1:** Bacterial strains and plasmids used in this study.

Bacterial strains	Description	Source
Marine Bacteria		
*V*. *coralliilyticus* OCN008	Responsible for tissue loss disease of the coral *Montipora capitata*; isolated in Kāneʻohe Bay, HI, USA; Ap^r^	[12]
*V*. *coralliilyticus* OCN014	Responsible for tissue loss disease of the coral *Acropora cytherea*; isolated from Palmyra Atoll; Ap^r^, Sm^r^	[15]
*V*. *coralliilyticus* ATCC BAA-450	Responsible for bleaching and tissue loss of the coral *Pocillopora damicornis*; isolated in Zanzibar; Ap^r^	[10]
*V*. *coralliilyticus* RE98	Responsible for mortalities of *C*. *gigas* larvae; isolated from a hatchery in Netarts, OR, USA; Ap^r^	[38]
*V*. *coralliilyticus* OCN008 ΔMSHA	Strain OCN008 with an in-frame, clean deletion of the mannose-sensitive type IV-gene cluster; severely reduced virulence in a coral infection model with *M*. *capitata*; Ap^r^	[15]
*V*. *coralliilyticus* OCN008 Δ*toxR*	Strain OCN008 with an in-frame, clean deletion of the *toxR* homolog; avirulent in a coral infection model with *M*. *capitata*; Ap^r^	[15]
*V*. *coralliilyticus* OCN008 Δ*ompU*	Strain OCN008 with an in-frame, clean deletion of the *ompU* homolog; Ap^r^	This study
*V*. *tasmaniensis* LGP32	Responsible for mortalities of adult *C*. *gigas*; isolated from the hemolymph of adult oysters collected along the Atlantic coast of France.	[43]
*Vibrio* spp. HMSC5	Non-virulent strain isolated during a larval mortality event at the Hatfield Marine Science Center in Newport, OR.	This study
*Alteromonas* sp. OCN004	Non-virulent strain isolated from healthy *M*. *capitata*; isolated in Kāneʻohe Bay, HI, USA.	[41]
***E*. *coli* strains**		
β3914	Conjugation strain; Δ*dapA*::(*erm*-*pir*); Km^r^, Em^r^, Tc^r^	[42]
π3813	Conjugation strain; Δ*thyA*::(*erm*-*pir*); Em^r^	[42]
**Plasmids**		
pACT3	P_tac_ expression vector; Cm^r^	[44]
pRK2013	Self-transmissible conjugation vector; Km^r^	[45]
pSW4426T	Suicide vector; Cm^r^, Sp^r^, Sm^r^	[42]
pBU231	Suicide vector used to delete *ompU* in OCN008; Cm^r^, Sp^r^, Sm^r^	This study
pBU246	P_tac_ expression vector for complementing deletion mutants; Cm^r^, Sp^r^, Sm^r^	This study
pBU248	Complementation vector expressing the wild-type copy of the *toxR* homolog from OCN008; Cm^r^, Sp^r^, Sm^r^	This study
pBU249	Complementation vector expressing the wild-type copy of the *ompU* homolog from OCN008; Cm^r^, Sp^r^, Sm^r^	This study

Abbreviations: Ap^r^ = resistance to ampicillin; Cm^r^ = resistance to chloramphenicol; Sp^r^ = resistance to spectinomycin; Sm^r^ = resistance to streptomycin; Km^r^ = resistance to kanamycin, Em^r^ = resistance to erythromycin; and Tc^r^ = resistance to tetracycline.

### Plasmid construction

All plasmids used in this study are listed in Table 1 and DNA oligonucleotides are listed in Table 2. Plasmid pBU231 is a suicide vector based on pSW4426T [42] that was used to cleanly delete all but the first 18 and last 18 nucleotides of the *ompU* coding region [ERB66165] in OCN008. Regions upstream and downstream of *ompU* were amplified from OCN008 chromosomal DNA by PCR with the primer pairs ompU-up-ClaI-F and ompU-up-OEX-R and ompU-down-OEX-F and ompU-down-SpeI-F, respectively. The up- and down-stream fragments were fused together by overlap extension PCR [46] then cloned as a *Cla*I/*Spe*I fragment into the same sites on pSW4426T to create pBU231. Plasmids were confirmed using PCR and Sanger sequencing using the primer pair pSW4426T-MCS-F and pSW4426T-MCS-R.

**Table 2:** DNA oligo nucleotides used in this study.

Primer	Primer sequence (5’ to 3’)	Source
pSW4426T-MCS-F	CTCAACGGGAATCCTGCTCTGCGAG	[15]
pSW4426T-MCS-R	ACTGCTTGGTGCCAGCCAATGAG	[15]
pSW4426T-cat-F	CGCCGGCCAGCCTCGCAGA	This study
pSW4426T-oriT-R	TTACGCCCCGCCCTGCCACTC	This study
pBU246-MCS-F	GTGCAGGTCGTAAATCACTGCATAA	This study
pBU246-MCS-R	CAGAGCAAGAGATTACGCGCAGACCA	This study
ompU-up-ClaI-F	ATATATATCGATATCACAGTCAATCAAAGTGACGGTT	This study
ompU-up-OEX-R	AGAAGTCGTAACGTAGGATCAGAGTTTTGTTCATCTTATAAGTCCTAA	This study
ompU-down-OEX-F	GAACAAAACTCTGATCCTACGTTACGACTTCTAATTTGTGACTTAAT	This study
ompU-down-SpeI-R	ATATATACTAGTACGCTGTTTAACATTCGTTGAGTGTAA	This study
Sp/Sm-EcoRV-F	ATATATGATATCAAAGGTCATTCAAAAGGTCATCCACCGGAT	This study
Sp/Sm-EcoRV-R	ATATATGATATCAAAGGTCATCCACCGGATCAGCTTAGTAAA	This study
pACT3-Cm-up	ACCTCTTACGTGCCGATCAACGTCTCAT	This study
pACT3-Cm-down	TATTGGTGCCCTTAAACGCCTGGTGCT	This study
pSW4499-cat-F	TTACGCCCCGCCCTGCCACTC	This study
pSW4499-oriT-R	CGCCGGCCAGCCTCGCAGA	This study
Vcor-toxR-SD-EcoRI-F	ATATATGAATTCTGATGAAATCTATACAACAATAATGGTCATATCCA	This study
Vcor-toxR-BamHI-R	ATATGGATCCCTATTGGCAAACTTTACTGAGGTCTTGCTG	This study
Vcor-ompU-SD-EcoRI-F	ATATATGAATTCGGACAGTAAATTAGGACTTATAAGATGAAC	This study
Vcor-ompU-SalI-R	ATATATGTCGACTTAGAAGTCGTAACGTAGACCTAGAGCT	This study

Plasmid pBU246 is a replicative TAC promoter (P_TAC_) expression vector derived from pACT3 [44] to genetically complement OCN008 deletion mutants. PCR with the primer pair pACT3-Cm-up and pACT3-Cm-down was used to remove the chloramphenicol resistance cassette from pACT3 and to linearize the plasmid. The spectinomycin/streptomycin (Sp/Sm) cassette from pRL1383a was cloned into the plasmid. The Sp/Sm resistance cassette was amplified from pRL1383a by PCR with the primer pair Sp/Sm-EcoRV-F and Sp/Sm-EcoRV-R. The PCR product was digested with *Eco*RV and ligated to the linearized pACT3 PCR product to create pBU237. The plasmid pSW4426T was used as template for PCR with the primers pSW4499-oriT-R and pSW4499-cat-F to amplify the *ori*T and the chloramphenicol resistance cassette. The PCR product was then cloned into the *Nru*I site of pBU237 to create plasmid pBU246.

Plasmid pBU248 is an expression plasmid to complement the *toxR* deletion mutant with a wild-type copy of the *toxR* homolog [ERB64497]. OCN008 DNA was used as template for PCR with the primers Vcor-toxR-SD-EcoRI-F and Vcor-toxR-BamHI-R. The PCR product was digested with *Eco*RI and *Bam*HI, then cloned into the same sites in pBU246 to create pBU248.

Plasmid pBU249 is an expression plasmid to complement the *ompU* deletion mutant with a wild-type copy of the *ompU* homolog [ERB66165]. OCN008 DNA was used as template for PCR with the primers Vcor-ompU-SD-EcoRI-F and Vcor-ompU-SalI-R. The PCR product was digested with *Eco*RI and *Sal*I, then cloned into the same sites in pBU246 to create pBU249.

### Bacterial conjugation and mutant creation

All plasmids were introduced in *V*. *coralliilyticus* using tri-parental conjugations with *E*. *coli* as previously described with slight modifications [12,15]. Donor and recipient strains were grown overnight with the appropriate antibiotics and DAP or DT as required. Overnight cultures were diluted 1:1000 in fresh culture medium without antibiotics, grown to an OD_600_ of 0.4, and then one ml was washed three times with either ASW or PBS for *V*. *coralliilyticus* or *E*. *coli* strains, respectively. The strains were then combined, resuspended in ASW to a total volume of 50 μl, and spotted onto GASW-DEX plates supplemented with DAP and DT. Conjugation spots were incubated at 30°C for 15 h before being resuspended in ASW, washed three times with ASW, diluted, and plated onto GASW-DEX supplemented with chloramphenicol, but lacking DAP or DT, and incubated at 27°C. Chloramphenicol-resistant colonies were streaked for isolation on GASW-DEX with spectinomycin and streptomycin, then colonies were screened for the presence of the replicative or suicide plasmid integrated into the chromosome using colony PCR and the primers pBU246-MCS-F and pBU246-MCS-R or pSW4426T-cat-F and pSW4426T-oriT-R, respectively. For strains with replicative plasmids for complementation, the presence of the plasmid was re-confirmed with PCR using pBU246-MCS-F and the reverse primer for the insert. For introduced suicide vectors, colonies of *V*. *coralliilyticus* with the integrated plasmid were grown for 15 h in GASW-DEX broth. Cultures were washed with ASW three times, diluted, and plated onto GASW-ARA to isolate mutants with a clean deletion of the target gene. Mutants were confirmed using PCR and primers specific to the gene being mutated.

### Pathology of *Vibrio*-challenged larvae

Seven-day old larval Pacific oysters, hatchery seawater, and algae (to serve as oyster feed) were provided by Joan Hendricks, Taylor Shellfish Farms, Quilcene, WA. They were shipped chilled (with ice packs) to the USDA laboratory in Dover, DE by overnight Federal Express. Upon arrival, seawater was filtered through a 0.22-μm pore size filter, aerated, quickly warmed to room temperature, and then the larvae were suspended in the seawater to approximately 50 larvae/ml. One-half ml was added to wells of ten 24-well flat bottom Corning-Costar plates (Corning Inc., Kennebunk, ME). Larvae were fed a ration of algae and five plates were incubated at 23°C while the remaining plates were incubated at 27°C overnight. The next day, overnight cultures of BAA-450, OCN008, OCN014 and RE98 were subcultured in Difco Luria Bertani broth (Baltimore Biological Laboratories, Sparks, MD) made with seawater at 30 ppt salinity and incubated to an optical density measured at 600 nm (OD_600_) of 0.80. Wells were inoculated with approximately 5 × 10^4^ CFU of each pathogen per ml of seawater and incubated at either 23 or 27°C. A negative control plate containing larvae without *V*. *coralliilyticus* was also maintained. Pathological effects of the strains to the larvae were determined at 24, 48 and 72 h post-challenge under an Eclipse TE200 inverted microscope (Nikon, Melville, NY) using a 60X objective. Representative images were captured at each time interval using a Nikon DS-Ri1 camera, a Nikon Digital Sight DS-U3 power supply and NIS Elements F4.30.00 digital software (Nikon).

### Oyster larvae infection trials

Oyster larvae were provided by Dr. Christopher Langdon and were spawned from captive breeding pairs at the OSU Hatfield Marine Science Center in Newport, OR. Larvae were maintained in seawater that had been adjusted to 35 ppm salinity, 25°C, and a pH between 8.1 and 8.25 (adjusted with calcium carbonate if necessary) and filtered through a 1-μm pore size filter. Concentrations of 72-h old *C*. *gigas* larvae were adjusted to approximately 40 larvae per ml using a cell strainer (45-μm pore size) and seawater previously filtered through a 0.2-μm membrane (FSW). The larval suspension was then equally distributed into a sterile 24-well plate with 500 μl of FSW containing roughly 20 larvae per well. Plates with larvae were kept in a humidified incubator to prevent evaporation and were fed daily with live algae (*Isochrysis* sp). Larvae were allowed to acclimate in their respective incubators for at least 24 h before any experimental treatment.

To prepare bacteria for virulence assays, glycerol stocks kept at -80°C were used to inoculate GASW broth that was then incubated overnight. The saturated cultures were diluted 1:100 in fresh GASW broth and then grown to an OD_600_ of 1.8, which corresponds to late-log phase. For strains with complementation plasmids, overnight cultures were grown in GASW with chloramphenicol and then subcultured into GASW supplemented with IPTG at a final concentration of 5 mM before being grown to late-log phase. The cells were then spun down and washed with FSW once, resuspended in FSW, and then 10-fold serial dilutions were made using FSW. The CFU/ml of the inocula was determined by plating dilutions of cultures on GASW agar and counting subsequent colonies. One plate of larvae (4 x 6 wells) contained a replicate of four wells (*n* = 4) for each bacterial treatment. Each treatment consisted of FSW, larvae and 10 μl of a bacterial suspension for final concentrations of 10^2^ through 10^6^ CFU of the appropriate bacterial cells/ml seawater, while the sixth set of wells was a control with larvae and FSW only. Plates were then incubated at the appropriate temperature for 72 h with daily feedings. After the incubation period, live and dead larvae were counted using an inverted microscope. Before enumeration, isoeugenol (0.4% v/v final concentration) was added to the wells to anesthetize the larvae without causing death.

### Corals and infection experiments

*Montipora capitata* fragments were collected under special activities permit #2018–03 issued by the Hawaiʻi Department of Land and Natural Resources, Division of Aquatic Resources by authorized individuals. Plating *M*. *capitata* fragments roughly 3 x 3 x 1 cm in size were collected from colonies on the fringing reef surrounding the island Moku o Loʻe (21.4339° N, 157.7881° W) in Kāneʻohe Bay, Hawaiʻi. Corals were allowed to recover in a flow-through seawater table at ambient light and temperature for at least 48 h before being used in infection experiments. Infection experiments were conducted at the University of Hawaiʻi at Mānoa as previously described [15]. This included the control bacterium *Alteromonas* sp. OCN004, an abundant culturable isolate from healthy *M*. *capitata* [41]. Infection trials utilized a block design with fragments from the same colony exposed to FSW, control bacterium OCN004, control bacterium HMSC5, the wild-type strain (OCN008), the Δ*toxR* strain, or the Δ*ompU* strain. A final concentration of 10^8^ CFU/ml of tank water was used for every strain of bacterium and water temperature was maintained at 27°C.

## Results

### Pathological changes of oyster larvae exposed to *V*. *coralliilyticus* strains

**Fig 1:**
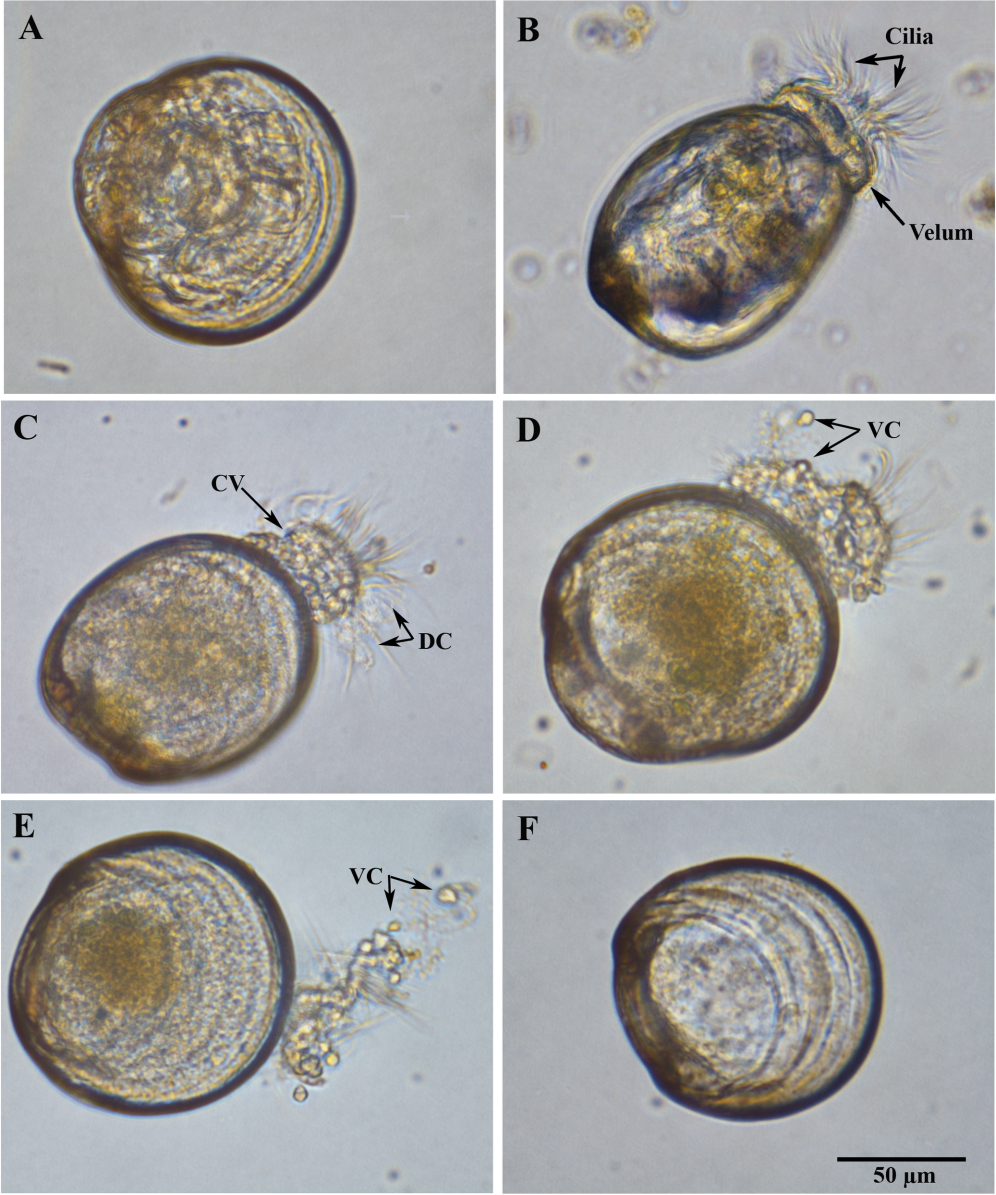
Pathology of Pacific oyster larvae before and after infection with *V*. *coralliilyticus* strain OCN014. (A) A sedentary, uninfected control larva with internal structures visible through the transparent shell and (B) actively swimming, uninfected larvae with smooth velum and ordered cilia. (C) A diseased larva with disfigured or clumped velum (CV) and disorganized cilia (DC). (D) Diseased larvae with release of the velar cells (VC) as the velum disassociates. (E) A diseased larva with substantially degraded velum and disorganized cilia. Most of the velar cells have been released into the seawater. (F) Dead larva without visible internal structures. The black scale bar represents 50 μm and applies to all images.

Larval oysters were challenged with *V*. *coralliilyticus* strains BAA-450, RE98, OCN008, and OCN014 and incubated at either 23 or 27°C for 72 h. Photomicrographs taken at 600X magnification (Fig 1A and 1B, captured after 24 h at 23°C) are representative of the uninfected control larvae, which maintained a normal appearance throughout the 72-h study at both 23 and 27°C. The larvae displayed typical coloration and distinct tissue organization of their organs that were partially visible through their transparent shell (Fig 1A). When motile, the velum protruded opposite the straight hinge (D-hinge stage larvae), and was normally round to ovoid, with a smooth contour, and abundant long, gently curved, regularly spaced cilia (Fig 1B). No differences were noted between the control larvae at 23 and 27°C, with the exception that the higher temperature caused an apparent increase in larval motility.

All four strains of *V*. *coralliilyticus*, at both observed temperatures and throughout the 72-h incubation, expressed identical pathologies. Examples of representative changes caused by OCN014 are shown in Fig 1C–1F. Early stages of infection show a clumped or disfigured velum (CV) and disorganized cilia (DC) (Fig 1C), which is in direct contrast to the smooth contours of the velum of the control larva in Fig 1B. At later stages, the velum became more disorganized and what appeared to be velar cells (VC) were released into the surrounding seawater as the velum degraded (Fig 1D). Degradation continued with the loss of additional velum mass via the release of VC (Fig 1E). This entire process from start to finish can occur overnight, depending on the concentration of *V*. *coralliilyticus* used. Larval death was clearly observed by the loss of distinguishable internal structures and the presence of an empty, relatively colorless shell (Fig 1F).

**Fig 2:**
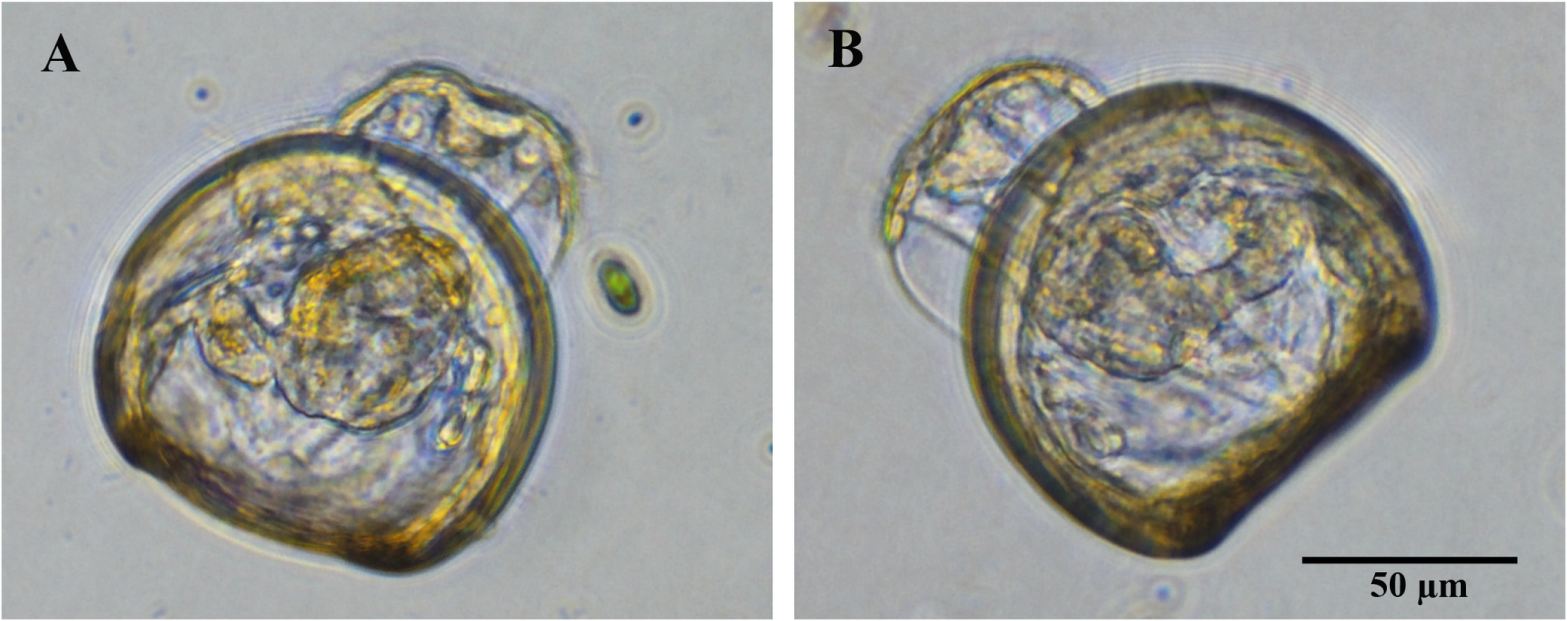
Protruding velum of oyster larvae maintained at elevated temperature. An unusual protruding velum (PV) in Pacific oyster larvae was occasionally noted after infection with all four *V*. *coralliilyticus* strains at 27°C. Pictured here are representative larvae infected with (A) RE98 and (B) OCN008 24 h post-inoculation. Scale bar is 50 μm and applies to both images.

The progression of larval disease also resulted in the loss of larval motility due to the degradation of the velum and cilia. Overall, the initiation of disease signs occurred sooner at 27°C than at 23°C. In addition, some of the *Vibrio*-infected larvae developed a protruding velum (Fig 2), which appears to be an occasional indicator of larval stress. A larva with a protruding velum typically had no cilia, so it exhibited no swimming motility, but was able to draw the protruding velum partially back within the valves of the oyster. It should be stressed that there were no clear morphological difference in larval pathologies with the four different *V*. *coralliilyticus* strains.

### The effect of temperature and *V*. *coralliilyticus* concentration on *C*. *gigas* mortalities

**Fig 3:**
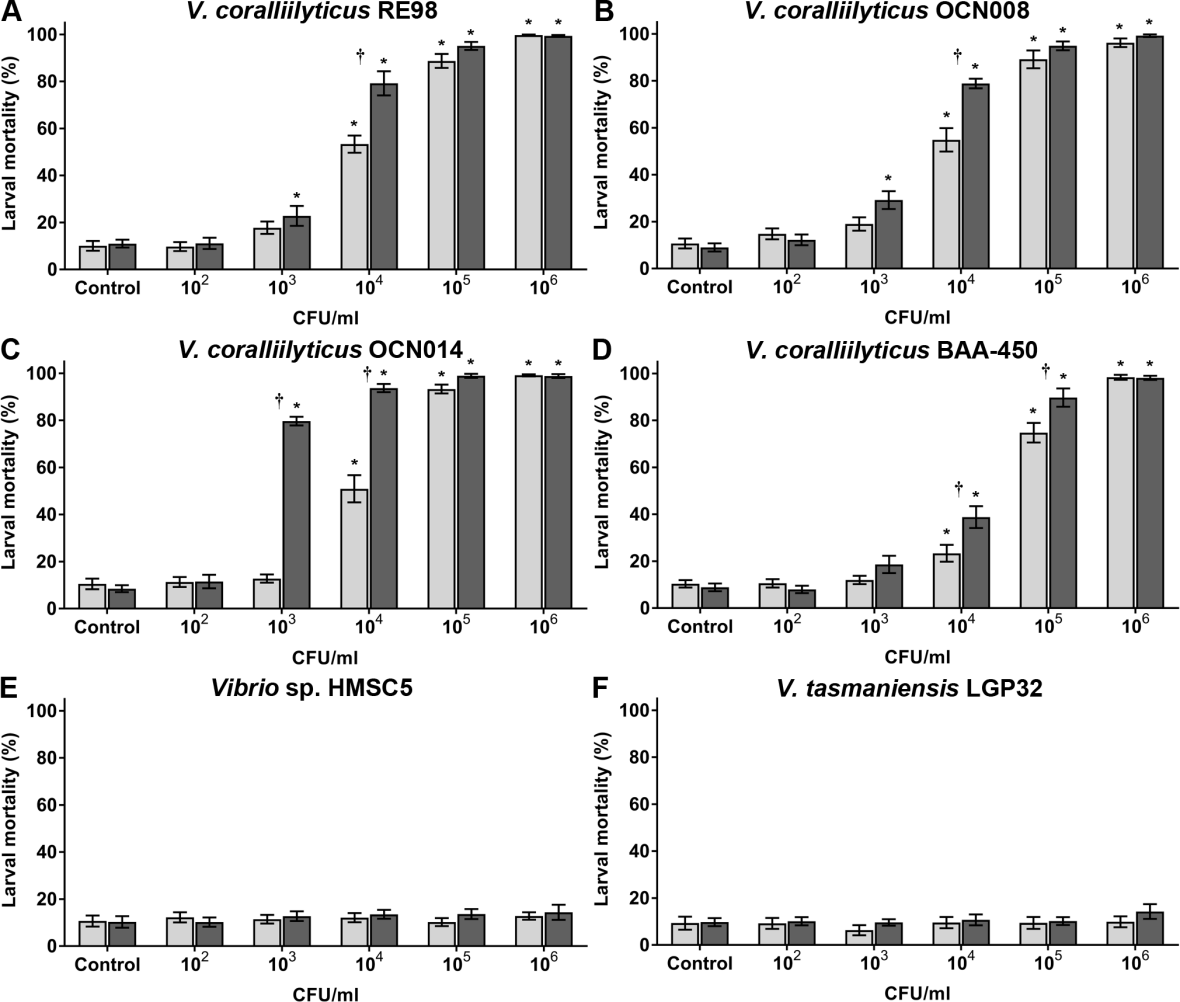
Percent mortality of oyster larvae 72 h post-inoculation with *V*. *coralliilyticus* strains. Larvae exposed to varying concentrations of *V*. *coralliilyticus* strain (A) RE98, (B) OCN008, (C) OCN014, (D) BAA-450, (E) *Vibrio* sp. strain HMSC5, and (F) *V*. *tasmaniensis* strain LGP32. Light gray bars represent the mean counts from larvae incubated at 23°C; dark gray bars represent larvae at 27°C. Control wells were inoculated with sterilized seawater. An asterisk (*) indicates that larval counts are significantly different from the controls (2-way ANOVA, *p* < 0.05, *n* = 12). A dagger (†) indicates the larval counts are significantly different for experiments at 23°C versus 27°C (2-way ANOVA, *p* < 0.05, *n* = 12).

All larval mortality data is shown in S1 Table. After exposure to the larval oyster pathogen RE98 at 23°C, a dosage of ~10^4^ CFU/ml of seawater killed ~50% of the larvae (Fig 3A and S1 Table). At a dose of 10^4^ CFU/ml there were significantly more larval mortalities (25.8% difference) in the wells exposed to RE98 at 27°C than in the 23°C-exposed wells (Tukey’s test, *p* < 0.01, *n* = 12). However, at 10^3^ and 10^5^ CFU/ml there was no significant difference in larval mortalities at 23°C and 27°C (Fig 3A). Exposure to OCN008 resulted in near-identical average larval mortalities to RE98 at 23 and 27°C after 72 h (Fig 3B and S1 Table). Similar to RE98, significantly more larvae died after exposure to OCN008 at 27°C compared to 23°C (23.9% difference) at a pathogen concentration of 10^4^ CFU/ml (Tukey’s test, *p* < 0.01, *n* = 12), but no difference was observed at the 10^3^−10^5^ CFU/ml treatments (Fig 3B).

Increased water temperatures had a more dramatic effect on OCN014 trials. At a pathogen concentration of 10^3^ and 10^4^ CFU/ml, there was significantly greater larval mortality at 27°C compared to larvae at 23°C (Tukey’s test, *p* < 0.01, *n* = 12), representing a 67% and 43% difference, respectively, (Fig 3C). For larvae exposed to OCN014 with 10^4^ CFU/ml at 23°C there was ~50% larval mortality, while at 27°C mortality was >90%. In comparison to RE98, OCN008 and BAA-450, strain OCN014 produced significantly higher levels of mortality (*p* < 0.01) compared to the other strains at a dosage of 10^3^ CFU/ml at 27°C (Fig 3, S1 Table).

For end-point mortality after 72 h, the least larval mortalities were observed with BAA-450 (Fig 3D and S1 Table), which was seemingly only virulent at concentrations ≥10^4^ CFU/ml. There were significantly more larval mortalities in the 10^4^ and 10^5^ CFU/ml treatments at 27°C compared to 23°C, a 15.4% and 14.9% difference, respectively (Tukey’s test, *p* < 0.01, *n* = 12). At 27°C, it required 10^5^ CFU/ml of water to cause >50% larval mortality, approximately 10-fold more than OCN008 or RE98 and 100-fold more than OCN014.

*Vibrio* sp. HMSC5 served as a negative bacterial control and did not have any noticeable negative effects on the oyster larvae (Fig 3E). Larvae were also exposed to the adult *C*. *gigas* pathogen *Vibrio tasmaniensis* [43,47], however, exposure to this bacterium also did not cause any significant levels of larval mortality (Tukey’s test, *p* > 0.9, *n* = 12) (Fig 2F). The water temperature did not influence larval survival (Tukey’s test, *p* > 0.9, *n* = 12 each treatment) (Fig 3E and 3F). Altogether, larvae were susceptible to infection and death from *V*. *coralliilyticus* RE98, OCN008, OCN014 and BAA-450 with OCN014 the most virulent and BAA-450 the least.

### Virulence of ToxR and MSHA deletion mutants in oyster larvae

**Fig 4:**
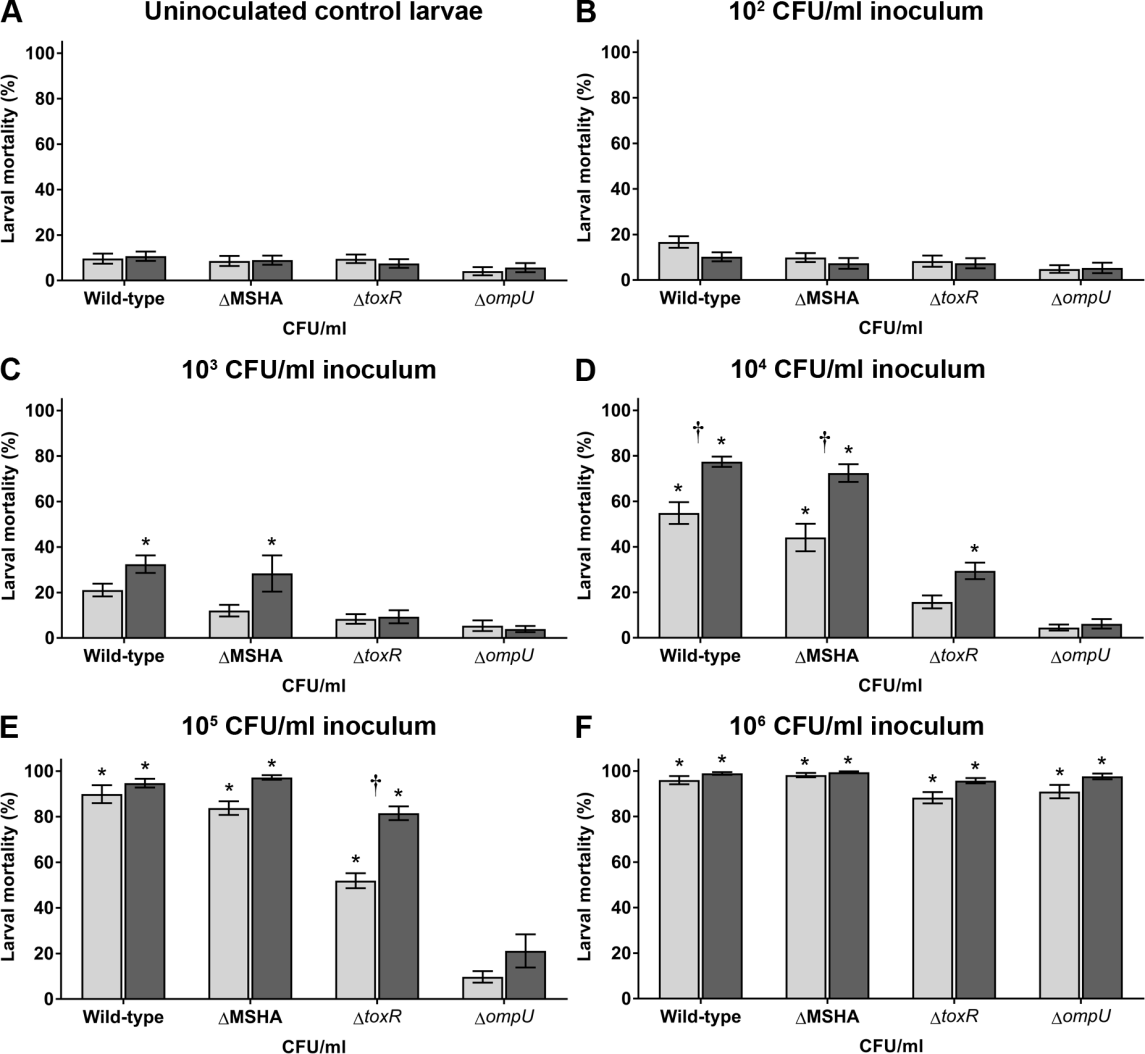
Percent mortality of *C*. *gigas* larvae exposed to OCN008 mutants not virulent to coral. Larvae were exposed to OCN008, the OCN008 ΔMSHA strain, the OCN008 Δ*toxR* strain, or the OCN008 Δ*ompU* strain. Control larvae were inoculated with (A) sterile seawater while test larvae were inoculated with strains of *V*. *coralliilyticus* to a final bacterial concentration of (B) 10^2^, (C) 10^3^, (D) 10^4^, (E) 10^5^, or (F) 10^6^ CFU/ml of seawater. Light gray bars represent the mean counts from larvae incubated at 23°C; dark gray bars represent larvae at 27°C. An asterisk (*) indicates larval counts significantly different from the uninoculated control averages (2-way ANOVA, *p* < 0.05, *n* = 12). A dagger (†) indicates the larval counts are significantly different for experiments at 23°C versus 27°C (2-way ANOVA, *p* < 0.05, *n* = 12).

**Fig 5:**
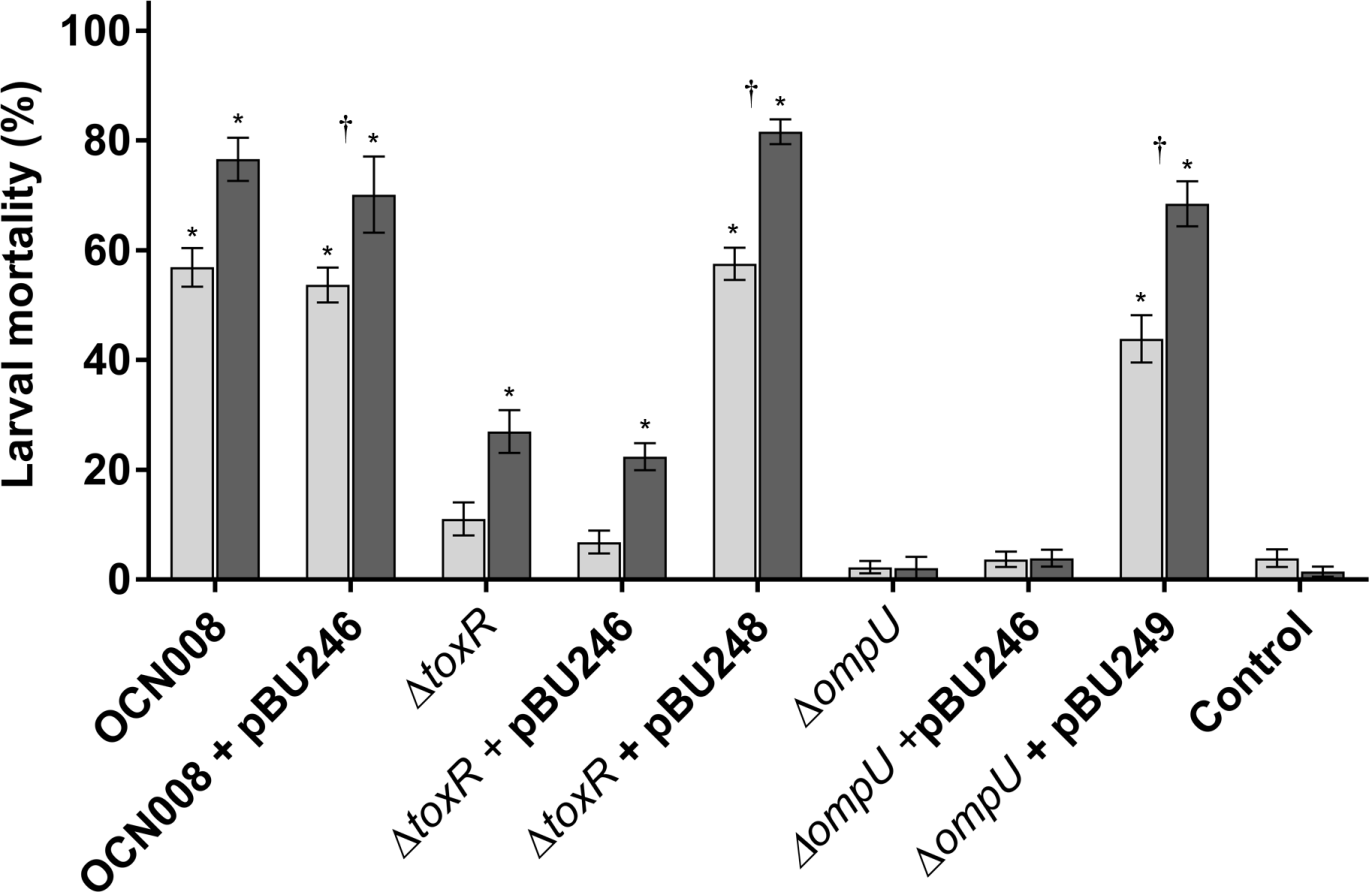
Percent mortality of oyster larvae exposed to genetically complemented OCN008 mutants. Larvae were exposed to 10^4^ CFU/ml of OCN008, OCN008 with the empty expression vector (pBU246), the Δ*toxR* strain, the Δ*toxR* strain with pBU246, the Δ*toxR* strain with the *toxR* complementation vector (pBU248), the Δ*ompU* strain, the Δ*ompU* strain with pBU246, or the Δ*ompU* strain with the *ompU* complementation vector (pBU249) for 72 h. Control larvae were inoculated with sterile seawater while test larvae were inoculated with strains of *V*. *coralliilyticus* to a final bacterial concentration of 10^5^ CFU/ml of seawater. Light gray bars represent the mean counts from larvae incubated at 23°C; dark gray bars represent larvae at 27°C. An asterisk (*) indicates larval counts significantly different from the uninoculated control averages (2-way ANOVA, *p* < 0.05, *n* = 12). A dagger (†) indicates the larval counts are significantly different for experiments at 23°C versus 27°C (2-way ANOVA, *p* < 0.05, *n* = 8).

ToxR and the MSHA type IV pili are important for coral infections in multiple *V*. *coralliilyticus* strains [15]; however, it was not yet known if infection of different hosts would require the same virulence factors. Therefore, previously created OCN008 mutants with in-frame deletions of *toxR* and the MSHA-encoding gene cluster, OCN008 Δ*toxR* (hereafter Δ*toxR*) and OCN008 ΔMSHA (hereafter ΔMSHA) [15], were tested in larval oyster infection assays. Virulence of the ΔMSHA strain was similar to the wild-type strain for each bacterial concentration and water temperature (Tukey’s test, *p* > 0.05, *n* = 12 each treatment), demonstrating that the MSHA pili are not required for infection of *C*. *gigas* larvae (Fig 4). In contrast, the Δ*toxR* strain demonstrated attenuated virulence compared to the wild-type strain; average larval mortality after exposure to the Δ*toxR* strain was significantly lower at inoculum concentrations from 10^4^ to 10^5^ CFU/ml compared to the wild-type strain (Tukey’s test, *p* < 0.05, *n* = 12 each treatment) (Fig 4). Interestingly, larval mortalities after exposure to the Δ*toxR* strain were still significantly higher at 27°C compared to 23°C when using a dose of 10^5^ CFU/ml (Tukey’s test, *p* < 0.01, *n* = 12 each treatment) (Fig 4E). In addition, the diseased larvae exposed to the Δ*toxR* strain displayed gross disease signs identical to those observed with the wild-type and ΔMSHA strain (data not shown). The Δ*toxR* strain was genetically complemented with the vector pBU248, restoring virulence to wild-type strain levels thus fulfilling Koch’s molecular postulates (Tukey’s test, *p* > 0.9, *n* = 8) (Fig 5). The empty expression vector, pBU246, did not influence virulence of the wild-type or Δ*toxR* strains. These results demonstrate that the MSHA pili, although important for coral infections, are not required for infection of *C*. *gigas* larvae; in contrast, ToxR, which is important for coral virulence, is also important for infection of oyster larvae.

### OmpU is essential for OCN008 infections of larval oysters and corals

In most vibrios, ToxR regulates a variety of proteins such as the outer membrane protein OmpU [28,48], a virulence factor for several pathogenic species [33,35,36]. Therefore, to investigate if the observed loss of virulence in the *toxR* mutant is attributed to reduced expression of OmpU, an OCN008 *ompU* deletion mutant (Δ*ompU*) was created and virulence was evaluated. The Δ*ompU* strain was avirulent to oyster larvae at a concentration ≤10^5^ CFU/ml at both 23 and 27°C (Tukey’s test, *p* < 0.01, *n* = 12) (Fig 4). Furthermore, temperature had no observable effect on larvae mortalities during trials with the Δ*ompU* strain. At a concentration of 10^6^ CFU/ml, larval mortalities were > 90%, however, this was observed with all other wild-type and mutant strains of *V*. *coralliilyticus* excluding the control vibrios HMSC5 and *V*. *tasmaniensis* (S1 Table). Furthermore, the loss of virulence observed for the Δ*ompU* strain could be genetically complemented with the vector pBU249 (Fig 5). Collectively, these experiments demonstrate that OmpU is essential for *V*. *coralliilyticus* infections of oyster larvae, while temperature does not affect this mutant to the same degree as the *toxR* mutant or wild-type strain.

**Fig 6:**
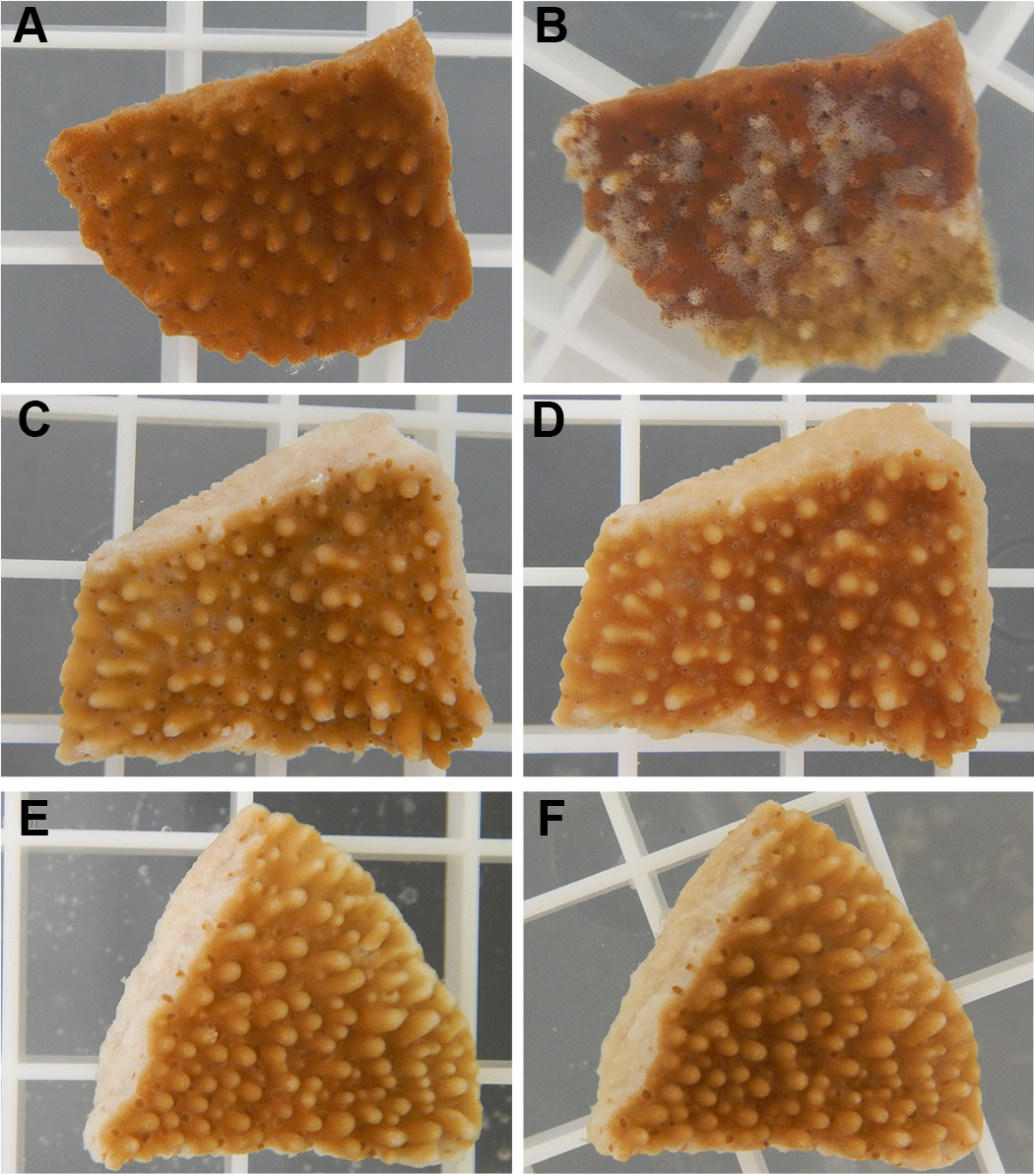
Representative photographs of *M*. *capitata* fragments used in infection experiments. (A) A coral fragment before exposure to OCN008, and (B) the same coral fragment 24 h post-exposure to OCN008 exhibiting extensive tissue loss/lysis. (C) A fragment before exposure to the Δ*ompU* strain of OCN008, and (D) the same fragment 240 h post-exposure to the Δ*ompU* strain. (E) A fragment before exposure to the control bacterium HMSC5, and (F) the same fragment 240 h post-exposure to HMSC5. The white square grating measures 1 x 1 cm.

OmpU appears to be essential for OCN008 infection of the coral species *M*. *capitata* (Fig 6). As expected, the wild-type strain caused extensive tissue loss in 87.5% of all exposed coral fragments (McNemar’s test, *p* = 0.23, *n* = 8), while the Δ*toxR* strain (photo not shown) was re-tested alongside these strains and was avirulent (McNemar’s test, *p* < 0.9, *n* = 8), consistent with a previous report [15]. The Δ*ompU* strain was avirulent to exposed corals under infectious conditions for OCN008 (McNemar’s test, *p* < 0.9, *n* = 8). Control fragments exposed to FSW or the control bacteria HMSC5 did not display any signs of disease during the 10-day experiment (Fig 6E and 6F). Coral experiments were limited by specimen availability and logistics, so more extensive experimental conditions or complemented strains were not tested. These results suggest that OmpU is required for wild-type levels of infections of coral and oyster larvae by *V*. *coralliilyticus*.

## Discussion

A recent study demonstrated that the coral pathogen *V*. *coralliilyticus* strain ATCC BAA-450 was virulent toward Pacific oyster larvae [37]; however, it was unknown if cross-host infections are specific to this strain or if *V*. *coralliilyticus* could be a generalist pathogen. This current study was able to demonstrate that multiple *V*. *coralliilyticus* strains pathogenic to coral can also cause acute larval oyster mortalities. The ability of all the tested *V*. *coralliilyticus* strains to cause *C*. *gigas* mortalities, albeit to varying degrees, indicates that the pathogenic strains of this species can infect multiple hosts. Additionally, we were able to determine that the genes encoding the predicted regulatory protein ToxR and its commonly regulated outer membrane protein OmpU are important for oyster larval infections. Furthermore, we showed that loss of OmpU results in an attenuated phenotype in coral infection, which might explain some of the observed role of ToxR during infection. These are the first cell-associated virulence factors identified in *V*. *coralliilyticus* that are important for cross-species infections.

Temperature-influenced mortalities of *C*. *gigas* larvae caused by *V*. *coralliilyticus* are similar to previous coral infection data [12,15,21], indicating there may be a core set of virulence factors utilized to infect either host. For all tested *V*. *coralliilyticus* strains, there was a general trend of increased larval oyster mortality at 27°C compared to 23°C, while there were no apparent differences in larval pathologies at either temperature. At 27°C, there was a higher incidence of larvae with unusually protruding vela (Fig 2), but these protruding vela are not believed to be directly caused by the higher temperature because optimal growth conditions for hatchery culture of larval Pacific oysters is reportedly 28°C [49]. Temperature appeared to have the greatest effect on larval mortalities when oysters were exposed to OCN014 and BAA-450, reminiscent of the coral infection data published on these two pathogens [15,21]. In contrast, the difference between the two temperatures for OCN008-associated oyster larvae mortalities were less apparent, similar to the marginal effect temperature has on OCN008-induced coral infections [12]. It should be noted that the optimal growth temperature for hatchery culture of Pacific oysters has been reported as 28°C [49], but as *V*. *coralliilyticus* are more virulent at higher temperature, our results suggest that sub-optimal temperature conditions should be considered for hatchery production in order to reduce larvae mortalities. Taken together, the similarities between the shellfish and coral infection results suggests conserved *V*. *coralliilyticus* virulence factors for two different hosts.

To further probe the possibility of overlapping virulence factors used by *V*. *coralliilyticus* to infect different hosts, we examined the virulence of OCN008 *toxR* and MSHA deletion mutants, which have attenuated virulence towards corals [15], against oyster larvae. The infection trials with the OCN008 ΔMSHA and Δt*oxR* strains support the notion that *V*. *coralliilyticus* may use different virulence factors for oyster and coral infections. The ΔMSHA strain had an approximately 60% reduction of virulence in a coral infection model [15]. By comparison, it was equivalent to the wild-type strain in a *C*. *gigas* larvae model. This demonstrates that the MSHA pili are important for coral infection, but not for infection of oyster larvae. For the human pathogen *V*. *cholerae*, the MSHA pili are important for adhesion to zooplankton and biofilm formation [50,51], but they are not required for human infections and it is the toxin-coregulated pili that are essential for colonization of the human intestine [29,52]. Thus, a similar scenario may be occurring with *V*. *coralliilyticus*, where the MSHA pili, which are involved with coral infections but not with larval oyster infections, serve different roles depending on the environment or host. Alternatively, it is possible that the free-swimming *C*. *gigas* larvae are actively consuming *V*. *coralliilyticus* from the water column, while the sessile *M*. *capitata* hosts require more adherence factors for colonization. Though, it should also be noted that oyster larvae are kept at much higher densities per volume of water during the infection experiments and in hatcheries compared to coral, which could also negate the need for the MSHA pili. However, follow up studies on the exact mechanisms of these identified virulence factors are required before any conclusions can be drawn.

In contrast to the ΔMSHA strain, exposure to the OCN008 Δ*toxR* strain resulted in fewer larvae mortalities compared to the wild-type strain at all temperatures and inoculum concentrations, suggesting that ToxR is important for the infection of oyster larvae. In conjunction with our previous work that demonstrated a *toxR* mutant was required for coral infection [15], ToxR seems to be a conserved virulence factor for both oyster larvae and corals. In terms of function, ToxR is a well-described transcriptional regulator of virulence factors for several pathogenic vibrios [28,30,31,48,53], therefore, it could be conjectured that ToxR serves a similar role in *V*. *coralliilyticus*. One well-studied protein that is part of the ToxR regulon is OmpU, which had been demonstrated to be positively regulated at the transcriptional level by ToxR in multiple vibrios [32,54,55]. The described ToxR-*ompU* regulatory relationship in other pathogenic *Vibrio* species suggests that the attenuated virulence of the OCN008 Δ*toxR* strain is due to the reduced expression of the OmpU porin.

As a virulence factor for other pathogenic vibrios, OmpU can serve as an adhesin and is important for resistance to antimicrobial compounds produced by host organisms [33,35,36,54,56,57]. For the adult *C*. *gigas* pathogen *V*. *tasmaniensis* strain LGP32, OmpU is essential for resistance to host-derived defensins and for adhesion/invasion of host hemocytes [36,56]. Curiously, LGP32 was discovered to be avirulent to *C*. *gigas* larvae, which may result from an adult *C*. *gigas* protein, *Cg*-EcSOD, being absent in larvae. *Cg*-EcSOD is an opsonin for OmpU-mediated phagocytosis and LGP32 invasion of hemocytes, but it is not present in *C*. *gigas* until metamorphosis from free-swimming larvae to settled juveniles (spat) [58]. This suggests that there is something intrinsic to *V*. *coralliilyticus* that allows it to infect *C*. *gigas* larvae in an OmpU-dependent manner, but not *V*. *tasmaniensis*. Interestingly, OmpU has also been implicated in host recognition for LGP32 [56], as well as the squid symbiont *V*. *fischeri* [59], but *V*. *coralliilyticus* utilizes this protein to infect *C*. *gigas* and *M*. *capitata*, two distinctly different hosts. It is tempting to speculate that while OmpU is involved with host recognition and specificity to certain organisms for some vibrios, this protein may serve a different function for *V*. *coralliilyticus*.

OmpU is one of the few identified cell-associated virulence factors essential for *V*. *coralliilyticus* infections of coral. However, it is possible that OmpU may serve an alternative function for coral infections as it does for oyster larvae infections. There is mounting evidence supporting the activation of the coral immune system in response to *V*. *coralliilyticus* [22,60–62] and the protective role of coral microflora [63–68], which suggests that this pathogen possesses countermeasures to these host defenses. For other pathogenic vibrios, the multi-functional protein OmpU is also involved with resistance to antibiotics and host-derived antimicrobial compounds such as polymyxin B and bile, respectively [54,57,69]. Similar to what is observed with *V*. *cholerae* mutants, the OCN008 Δ*toxR* and Δ*ompU* strains are unable to grow on the bile salt-containing TCBS agar (Ushijima, per. observation), though, it is unclear if these strains are more susceptible to the coral immune system or antagonistic interactions with the host microflora. However, the presence of OmpU may not be the sole determinant for virulence towards corals. All four strains of *V*. *coralliilyticus* described here possess OmpU homologs with >90% amino acid similarity to each other (data not shown), but previous studies have demonstrated that OCN014 is significantly less virulent towards *M*. *capitata* compared to OCN008 [15]. At this stage, these interpretations are speculative, although, they do suggest that novel virulence factors for *V*. *coralliilyticus* should also be investigated for their role in overcoming host defenses.

An interesting phenomenon observed with all mutant strains during this study was the acute rise in larval mortalities when approximately 10^6^ CFU/ml of inoculum was used (Fig 4F). Why would virulence for the *ompU* mutant be attenuated at most concentrations but result in >80% motility at the highest doses tested? One possible explanation is the production of the previously described extracellular virulence factor, VtpA, a zinc-metalloprotease that is toxic to *C*. *gigas* larvae [70]. The cell-free culture supernatants from the Δ*toxR* and Δ*ompU* strains are toxic to *C*. *gigas* larvae like the wild-type strain, while immunological and genomic analysis indicated that OCN008 produces the VtpA protease (Ushijima and Häse, unpublished data). Furthermore, previous studies have determined that VtpA production increases with cell density and is positively regulated by the quorum sensing regulatory protein VtpR [70,71]. Therefore, OCN008 cultures grown to mid-exponential phase (OD_600_ = 1.6 to 1.8) and inoculated at a density of 10^6^ CFU/ml would theoretically produce more toxic exoenzymes such as VtpA, while lower doses would potentially need to replicate (through infection of *C*. *gigas* larvae) before lethal concentrations of toxins are present. As of now, this scenario is purely speculative, however, the dynamics between the cell-associated and extracellular virulence factors during infection warrants further investigation.

Taken together, this study demonstrates that several pathogenic *V*. *coralliilyticus* strains can cause acute mortalities of Pacific oyster larvae and that ToxR and OmpU are important for infection of both larval oysters and corals, while MSHA is only required for infection in corals. In conclusion, this study answers fundamental questions regarding *V*. *coralliilyticus* infectivity in corals and larvae under different dosage levels and temperature regimes and demonstrates that ToxR and OmpU contribute to their virulence.

## Supporting information

S1 Table Average *C*. *gigas* larvae percent mortalities 72 h post-inoculation. (DOCX)
