# Supplementary material for: Factors affecting infection of corals and larval oysters by Vibrio coralliilyticus
Source: PLoS One. 2018 Jun 19;13(6):e0199475. doi: 10.1371/journal.pone.0199475 (PMC6007914; doi:10.1371/journal.pone.0199475)
Supplement: S1 Table — (DOCX) [file pone.0199475.s001.docx]

**S1 Table. Average *C. gigas* larvae percent mortalities 72 h post-inoculation.**

| Strain and dosage (CFU ml^-1^) | At 23°C | | At 27°C | |
| --- | --- | --- | --- | --- |
|  | Mean mortality (%), *n*=12 | Standard error of the mean (%) | Mean mortality (%), *n*=12 | Standard error of the mean (%) |
| *V. coralliilyticus* RE98 |  |  |  |  |
| Control | 10.0 | 2.1 | 11.0 | 1.7 |
| 10^2^ | 9.8 | 1.9 | 11.1 | 2.4 |
| 10^3^ | 17.8 | 2.6 | 22.8 | 4.2 |
| 10^4^ | 53.3 | 3.7 | 79.2 | 5.1 |
| 10^5^ | 88.7 | 2.9 | 95.1 | 1.7 |
| 10^6^ | 99.7 | 0.3 | 99.4 | 0.4 |
| *V. coralliilyticus* OCN008 |  |  |  |  |
| Control | 10.7 | 2.1 | 9.0 | 1.7 |
| 10^2^ | 14.8 | 2.3 | 12.2 | 2.3 |
| 10^3^ | 19.0 | 2.9 | 29.2 | 3.8 |
| 10^4^ | 54.9 | 5.0 | 78.8 | 2.1 |
| 10^5^ | 89.1 | 3.8 | 94.9 | 1.9 |
| 10^6^ | 96.2 | 1.9 | 99.2 | 0.5 |
| *V. coralliilyticus* BAA-450 |  |  |  |  |
| Control | 10.4 | 1.6 | 8.8 | 1.6 |
| 10^2^ | 10.6 | 1.8 | 8.0 | 1.6 |
| 10^3^ | 12.0 | 1.8 | 18.6 | 3.7 |
| 10^4^ | 23.4 | 3.6 | 38.8 | 4.6 |
| 10^5^ | 74.8 | 4.2 | 89.7 | 3.9 |
| 10^6^ | 98.4 | 1.1 | 98.1 | 0.9 |
| *V. coralliilyticus* OCN014 |  |  |  |  |
| Control | 10.5 | 2.2 | 8.5 | 1.5 |
| 10^2^ | 11.3 | 2.1 | 11.5 | 2.9 |
| 10^3^ | 12.8 | 1.7 | 79.7 | 1.8 |
| 10^4^ | 50.9 | 5.8 | 93.7 | 1.7 |
| 10^5^ | 93.3 | 1.9 | 98.9 | 0.8 |
| 10^6^ | 99.1 | 0.5 | 98.8 | 0.8 |
| *Vibrio* sp. HMSC5 |  |  |  |  |
| Control | 10.7 | 2.4 | 10.3 | 2.5 |
| 10^2^ | 12.3 | 2.2 | 10.2 | 2.0 |
| 10^3^ | 11.5 | 1.9 | 12.8 | 2.0 |
| 10^4^ | 12.1 | 1.9 | 13.5 | 1.9 |
| 10^5^ | 10.2 | 1.7 | 13.7 | 2.1 |
| 10^6^ | 12.8 | 1.6 | 14.4 | 3.2 |
| *V. tasmaniensis* LGP32 |  |  |  |  |
| Control | 9.3 | 2.8 | 9.7 | 1.7 |
| 10^2^ | 9.2 | 2.3 | 10.1 | 1.7 |
| 10^3^ | 6.3 | 2.2 | 9.6 | 1.4 |
| 10^4^ | 9.5 | 2.4 | 11.0 | 2.3 |
| 10^5^ | 9.4 | 2.5 | 10.2 | 1.7 |
| 10^6^ | 9.9 | 2.3 | 14.2 | 3.2 |
| *V. coralliilyticus* OCN008 ΔMSHA |  |  |  |  |
| Control | 8.6 | 2.2 | 9.0 | 2.0 |
| 10^2^ | 9.9 | 2.0 | 7.3 | 2.4 |
| 10^3^ | 12.0 | 2.6 | 28.4 | 8.0 |
| 10^4^ | 44.1 | 6.0 | 72.4 | 3.9 |
| 10^5^ | 83.8 | 3.0 | 97.2 | 1.0 |
| 10^6^ | 98.2 | 0.9 | 99.4 | 0.4 |
| *V. coralliilyticus* OCN008 Δ*toxR* |  |  |  |  |
| Control | 9.6 | 1.9 | 7.5 | 1.9 |
| 10^2^ | 8.3 | 2.4 | 7.4 | 2.2 |
| 10^3^ | 8.4 | 2.1 | 9.3 | 2.9 |
| 10^4^ | 15.8 | 2.8 | 29.4 | 3.6 |
| 10^5^ | 51.9 | 3.3 | 81.5 | 3.0 |
| 10^6^ | 67.7 | 10.7 | 95.8 | 1.2 |
| *V. coralliilyticus* OCN008 Δ*ompU* |  |  |  |  |
| Control | 4.1 | 1.8 | 5.7 | 2.0 |
| 10^2^ | 4.9 | 1.7 | 5.3 | 2.3 |
| 10^3^ | 5.4 | 2.3 | 3.9 | 1.3 |
| 10^4^ | 4.5 | 1.3 | 6.1 | 2.1 |
| 10^5^ | 9.7 | 2.5 | 21.1 | 7.3 |
| 10^6^ | 91.0 | 2.9 | 97.6 | 1.3 |
